# Assessing farm biosecurity and farmers’ knowledge and practices concerning antibiotics and antibiotic resistance in poultry and pig farms in Southern Togo

**DOI:** 10.14202/vetworld.2022.1727-1737

**Published:** 2022-07-21

**Authors:** Andre Pouwedeou Bedekelabou, Essodina Talaki, Koffi Francois-Xavier Dzogbema, Malibida Dolou, Madi Savadogo, Malik Orou Seko, Rianatou Bada Alambedji

**Affiliations:** 1 Department of Public Health and Environment, Inter-States School of Veterinary Sciences and Medicine of Dakar (EISMV), P.O. Box 5077, Dakar, Senegal; 2 Higher School of Agronomy (ESA) of the University of Lomé, Togo; 3 Regional Center of Excellence on Avian Sciences (CERSA) of the University of Lomé, Togo; 4 Department of Biomedical and Public Health, Research Institute for Health Sciences (IRSS/CNRST), P.O. Box 7047, Ouagadougou, Burkina Faso; 5 Fundamental and Applied Research for Animals and Health, Faculty of Veterinary Medicine, University of Liege, Quartier Vallée 2 avenue de Cureghem, 6, 4000 Liege, Belgium

**Keywords:** antibiotic resistance, antibiotics, biosecurity, knowledge, pig, poultry, practice

## Abstract

**Background and Aim:**

Several factors contribute to the unusual incidence of antibiotic resistance, which is now a primary public health concern. However, failure in managing preventive and therapeutic antibiotic use on farms is one of the most crucial factors. Therefore, this study aimed to evaluate the biosecurity of farms, farmers’ competence, and practices related to antibiotics and their resistance in poultry and pig rearing in Togo.

**Materials and Methods:**

Through a cross-sectional survey, 121 commercial poultry farmers and 97 commercial pig farmers were questioned to evaluate the biosecurity of farms and farmers’ competence and practices related to antibiotics and antibiotic resistance. Descriptive analyses, including the evaluation of proportions, were carried out. In addition, results from qualitative factors were evaluated in a defined grid and totaled up to assess cleanliness measures, awareness, and behavior regarding antibiotics and their resistance.

**Results:**

The results demonstrated that most farmers working on poultry farms had a university education, while most working on pig farms had secondary education. Most poultry (69%) and pig (44%) farms were of small sizes (<1000 animals in poultry and <10 animals in pig farming). The footbaths were used in just 51% of poultry farms and 4% of pig farms, respectively, with 37% and 82% of poultry and pig farms having inadequate levels of hygiene. In poultry farms, respiratory issues and periodic decline in egg-laying were the main problems. Simultaneously, skin disorders (scabies) and plagues (African swine fever) were the primary health constraints in pig farming. Tetracycline is the most commonly used antibiotic by farmers. However, in poultry and pig farms, 21% and 67% of farmers were unaware of antibiotics. In addition, 39% and 57% were unaware of antibiotic resistance. Poultry and pig farmers’ competence were substantially linked to their education level. Poultry farmers demonstrated better practices, including procuring antibiotics based on veterinary prescriptions (63%) and they knew where antibiotics should be bought (90%). Nevertheless, 43% of farmers asserted unpleasant activities – no application for laboratory testing (93%) and use of antibiotics for prevention (82%). In pig farming, most farmers (69%) reported inadequate incidents of the use of antibiotics.

**Conclusion:**

This study identified a crucial non-compliance with biosecurity measures and good practices toward antibiotic use on many farms. Therefore, training of farmers is mandatory for safe livestock products.

## Introduction

Livestock is one of the fastest-growing agricultural areas, possibly providing economic growth and poverty alleviation prospects in rural communities. Nevertheless, there may be harmful outcomes to the development of this sector. The public health consequences of livestock production have recently become a source of concern [1]. There is the rapid growth of pathogenic diseases and the impact on the development and spread of antibiotic resistance. The growing use of antibiotics in livestock stems from the fact that small-scale farming is progressively replaced by intensive, large-scale, and specified commercial farming [2]. Large-scale and specialized commercial farms sprouting primarily around big cities to meet the growing demand for animal source proteins are recognized for facilitating global antimicrobial ingestion [3]. In animals, antibiotics treat clinical diseases, inhibit and regulate common disease events, and improve growth. Therefore, varying applications of antibiotics in animal feed have been described as therapeutic, prophylactic, and subtherapeutic [4].

Despite the broad adoption of antibiotic application in the food of animals and the proof that the application of antibiotics in animal feed production constitutes the principal causative factor to the ongoing antibiotic resistance predicament, reliable data on the range and configurations of use are lacking or unavailable for low- and middle-income countries [5]. It is interesting that Africa as a continent perhaps lacks information on antibiotic consumption [6]. The absence of monitoring systems for antibiotic use and resistance has been observed in several countries in the human and animal health sectors [7]. Nevertheless, the current information indicates increased levels of antimicrobial use in livestock [8, 9] with antibiotic abuse by farmers or animal health professionals [10, 11].

As a low- and middle-income country, Togo is going through rapid modernization of its livestock sector in reaction to the plea of large cities like Lomé, the administrative capital. This new modernization is more noticeable in poultry and pig farms and is preferred by private organizations usually looking for profit after investment. However, there are nearly no principles overseeing the modern commercial farms in the country, and the number of organizations is unknown since there is no mapping of the farms [12]. Furthermore, even though legislation on veterinary pharmacy exists, it is not enforced and illegal sales outlets of veterinary drugs prevail [13]. The dangers of non-compliance with manufacturing rules, such as concern for the environment and preservation of consumer health, may surface if these organizations are unaware and not monitored [14].

To the best of our knowledge, no assessment of hygiene management on farms and antibiotic application in modern poultry or pig farms has been examined to date. Therefore, this study was conducted to evaluate for the first time the biosecurity in farms and farmers’ competence and practices regarding the use of antibiotics.

## Materials and Methods

### Ethical approval and informed consent

Before each interview, the aim of the study was explained to farmers to get their consent in verbal form. They were informed of the possibility of refusing to participate at any time in the study by suspending the interview. Likewise, the breeders were guaranteed anonymity during data processing. Institutional approval was also granted from the Ministry of Agriculture through the service in charge of animal health.

### Study period, area, and survey

A cross-sectional survey was conducted from August to September 2019 in the first region (maritime) and from October to November 2020 in the second region (Plateau). Maritime and Plateau regions are two regions located on South Togo. The Maritime and Plateau regions are responsible for 65% of the country’s population (approximately 4.3 million inhabitants). These regions have various livestock production techniques for animal rearing, including large, modern poultry, and pig farms. They are sectors of excellence for modern poultry and pig rearing with more than 80% of the modern poultry farms and 40% of national pig production happening there [15]. For this study, only farmers with animals (poultry and pigs) on their farms at the time of the survey were included in the study. The participants were selected based on a respondent-driven process. The first respondents were chosen with the guidance of a local private veterinarian as a key informant. Based on the list of breeders suggested by the private veterinarian, the first category of breeders was interviewed. Then, breeders were asked to suggest the names of other farmers in the same community. The objective was to interview the maximum possible breeders, if not all. Finally, 121 poultry and 97 pig farmers were contacted regarding their accessibility and availability to participate in the study.

### Data collection

A literature review was carried out to guide the creation of the questionnaire. The developed questionnaire comprised both closed- and open-ended questions spanning general information about the farms (type of farm, updated number of animals, and health management), farmers (gender, education level, main occupation, and training), antibiotic use for the prevention of infectious diseases, the origin of antibiotics, farmer’s competence, and practices concerning antibiotic resistance. In addition, interviewers competent in poultry and pig farming and data collection conducted face-to-face interviews at the farms. Local languages were used for interviews when the farmers struggled with French. Interviewers completed the questionnaire based on the responses received to each question from the farmers.

### Assessment of hygiene, knowledge, and practices on farms

Questions were asked to evaluate, on the one hand, hygiene practiced on farms and, on the other hand, competence and practices related to antibiotics and antibiotic use. The answers were scored from 0 to 1: Zero, for bad practice/response, and one, for good practice/response. Adding up the given scores allowed us to rank the level of hygiene, competence, and farm practices. The measure of hygiene and practices regarding appropriate antibiotic use were then evaluated on a scale ranging from 0 to 5 – unsatisfactory, if the total scores ranged from 0 to 2, satisfactory, if the total scores ranked from 3 to 5. Finally, the farmers’ knowledge regarding antibiotics was evaluated on a scale ranging from 0 to 3 – unsatisfactory, if the total scores ranked from 0 to 1 and satisfactory, if the total scores ranked from 2 to 3.

###  Typifying multiple corresponding analyses and hierarchical classification for farms

Surveyed farmers were numbered 1–121 in the poultry database and 1–97 in the pig database following the interview rank. In the poultry database, Maritime farmers were enumerated from 1 to 70 and those from the Plateau were from 71 to 121. In the pig database, those from the Maritime were numbered from 1 to 46 and those from the Plateau were numbered from 47 to 97. Rx64 version 3.6.1 (package FactoMineR, functions multiple correspondence analysis [MCA] and hierarchical clustering, https://cran.r-project.org/bin/windows/base/old/3.6.1/) was used to conduct multiple correspondence analysis (MCA) and hierarchical classification analysis (HCA). MCA is a statistical technique used to evaluate the association among a set of qualitative variables. This technique projects the variable modalities on maps based on their correlations. Thus, the relationship between the modalities associated was established using MCA. The HCA is based on the MCA results to project each breeder according to their modalities on an area map defined by a virtual axis. This algorithmic technique permits defining hierarchically discrete groups (clusters) following the branches of a dendrogram. Therefore, the HCA interpretation is based on identical breeder modalities in the same cluster. This study used 24 variables with 50 modalities to demonstrate a typology of livestock breeders.

### Statistical analysis

The data extracted with Kobo Toolbox were transferred into a Microsoft Excel spreadsheet for statistical analysis. First, descriptive analyses, including the evaluation of ratios, were conducted. In addition, responses from qualitative factors were scored in a defined grid and added up to evaluate hygiene, knowledge, and practices regarding antibiotics and antibiotic resistance. Second, a Chi-square analysis was conducted in Statistical Package for the Social Sciences to evaluate the relationship between the dependent variables (knowledge of antibiotics and antibiotic resistance, practice regarding antibiotic resistance) and explanatory variables linked to breeder and farm behavior. Finally, R software v.4.1.2 (https://cran.r-project.org/) was used with the correct packages for multiple corresponding analyses and hierarchical classification.

## Results

### Characteristics of the surveyed farms and breeders

In poultry farms, the farmers were predominantly male (87%) and some farmers had a university education (52%). Breeding was a minor activity for most respondents (63%). A total of 61% of farmers asserted to have benefited from specific poultry farming training (Table-1). Among pig farmers, 68% of the respondents were male. However, the main level of education was secondary education (54%), and the majority did not receive specialized training in pig farming (73%). Concerning the hygiene aspect, 37% and 82% of poultry and pig farms, respectively, had an unsatisfactory level of hygiene.

**Table 1:** Characteristics of surveyed breeders in poultry and pig farms.

Poultry farmers		Study area		Repartition per region	
		
Variables	Modalities	Number of respondents (n = 121)	Frequency (%+CI)	Maritime (%+CI/n = 70)	Plateau (%+CI/n = 51)
Gender	Men	105	87 ± 6%	58 ± 9.4%	42 ± 9.4%
	Women	16	13 ± 6%	56 ± 24.3%	44% ± 24.3%
Education	Non-instructed	2	2 ± 2.3%	50 ± 49%	50 ± 49%
	Primary and secondary level	56	46 ± 8.9%	70 ± 1.6%	30 ± 1.6%
	University level	63	52 ± 8.9%	48 ± 1.6%	52 ± 1.6%
Main occupation	Breeding	51	46 ± 9%	51 ± 1.9%	49 ± 1.9%
	Other*	70	63 ± 9%	63 ± 1.4%	37 ± 1.4%
Training in poultry farming	Yes	74	61 ± 8.7%	58 ± 1.3%	42 ± 1.3%
	No	47	39 ± 8.7%	57 ± 2.1%	43 ± 2.1%

**Pig farmers**		**Number of respondents (n = 97)**	**Proportion (%+CI)**	**Maritime (%+CI/n = 46)**	**Plateau (%+CI/n = 51)**

Gender	Men	66	68 ± 9%	53.03 ± 12%	47 ± 12%
	Women	31	32 ± 9%	35.48 ± 17%	65 ± 17%
Education	Non-instructed	27	28 ± 9%	55.56 ± 19%	44 ± 19%
	Primary and secondary level	52	54 ± 10%	48.08 ± 14%	52 ± 14%
	University level	18	19 ± 8%	33.33 ± 22%	67 ± 14%
Main occupation	Breeding	46	44 ± 10%	41.30 ± 14%	53 ± 14%
	Other*	51	56 ± 10%	52.94 ± 14%	47 ± 14%
Training in pig’s farming	Yes	26	27 ± 9%	61.54 ± 19%	38 ± 19%
	No	71	73 ± 9%	42.25 ± 11%	58 ± 11%

* Cited occupation included government employment (teacher, physician…), self-employment (trader, builder, carpenter…). CI = Confidence interval

### Farms’ characteristics

In Table-2, parts A and B describe the features of poultry and pig farms. Overall, poultry was modern farming (commercial farms with exotic breeds) (77%; n = 93). In pig farming, enhanced traditional farms (commercial farms with home-grown breeds) formed the majority in the study community (72%; n = 70). The farms were mostly permanent producing farms (71% and 73% in poultry and pig farms, respectively). Most poultry (69%) and pig (44%) farms were small size farms (<1000 animals in the poultry and <10 animals in the pig farms).

**Table 2:** Characteristics of poultry and pig farms and hygiene practices of farmers.

A – Hygiene in poultry farms	Satisfactory (n = 76/62.8%)	Unsatisfactory (n = 45/37.2%)	p-value
Region			
Maritime (n = 70)	50 (71.4%)	20 (28.6%)	0.024
Plateau (n = 51)	26 (51.0%)	25 (49.0%)	
Gender			
Male (n = 105)	67 (63.8%)	38 (36.2%)	0.560
Female (n = 16)	9 (56.3%)	7 (43.8%)	
Education			
Non-instructed (n = 2)	0 (0.0%)	2 (100%)	0.103
Primary and secondary level (n = 51)	33 (58.9%)	23 (41.1%)	
University level (n = 63)	43 (68.3%)	20 (31.7%)	
Training			
Yes (n = 74)	49 (66.2%)	25 (33.8%)	0.331
No (n = 36)	27 (57.4%)	20 (42.6%)	
Type of farm			
Modern (93)	68 (73.1%)	25 (26.9%)	0.000
Improved traditional farm (28)	8 (28.6%)	20 (71.4%)	
Farm size			
Small (<1000)	48 (57.1%)	36 (42.9%)	0.049
Intermediate (1000–5000)	21 (70.0%)	9 (30.0%)	
Big size (>5000)	7 (100%)	0 (0.0%)	
Seniority of the farm			
Recent (<10 years)	61 (64.2%)	34 (35.8%)	0.648
Old (>10 years)	15 (57.7%)	11 (42.3%)	
Regularity of the production			
Permanent production (n = 87)	53 (60.9%)	34 (39.1%)	0.491
Temporary production according to mean availability (n = 34)	23 (67.2%)	11 (32.4%)	

**B – Hygiene in pig farms**	**Satisfactory (n = 17/15.5%)**	**Unsatisfactory (n = 80/82.5%)**	**p-value**

Region			
Maritime (n = 46)	8 (17.4%)	38 (82.6%)	0.974
Plateau (n = 51)	9 (17.6%)	42 (82.4%)	
Gender			
Male (n = 66)	15 (22.7%)	51 (77.3%)	0.049
Female (n = 31)	2 (6.5%)	29 (93.5%)	
Education			
Non-instructed (n = 27)	1 (3.7%)	26 (96.3%)	0.000
Primary and secondary level (n = 52)	7 (13.5%)	45 (86.5%)	
University level (n = 18)	9 (50%)	9 (50%)	
Training in farming			
Yes (n = 26)	10 (38.5%)	16 (61.5%)	0.002
No (n = 71)	7 (9.9%)	64 (90.1%)	
Type of farm			
Modern (27)	12 (44.4%)	15 (55.6%)	0.000
Improved traditional farm (70)	5 (7.1%)	65 (92.9%)	
Farm size			
Small (<10) (n = 43)	3 (7%)	40 (93%)	0.042
Intermediate (10–50) (n = 45)	11 (24.4%)	34 (75.6%)	
Big size (>50) (n = 9)	3 (33.3%)	6 (66.7%)	
Seniority of the farm			
Recent (<10 years) (n = 69)	15 (21.7%)	54 (78.3%)	0.072
Old (>10 years) (n = 28)	2 (7.1%)	26 (92.9%)	
Regularity of the production			
Permanent production (n = 71)	12 (16.9%)	59 (83.1%)	0.789
Temporary production (n = 26)	5 (19.2%)	21 (80.8%)	

Regarding hygiene and prevention measures, 82% of poultry farmers used a preventive register versus 29% of pig farmers. Routine disinfection of the farm (washing of the building with antiseptic products) was conducted by 89% of poultry farmers, while only 36% of pig farmers routinely disinfected their farms. Footbaths were recorded in 51% of poultry farms, while it was only 4% in pig farms. Finally, 37% and 82% of poultry and pig farms, respectively, had substandard hygiene measures.

**Figure 1:**
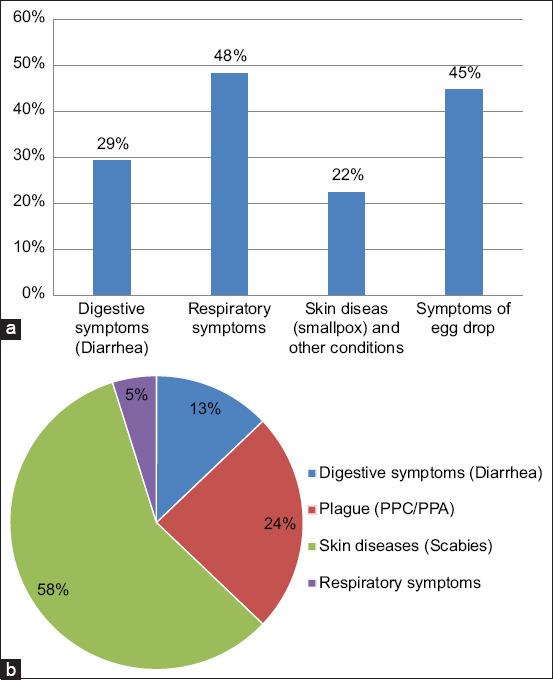
[Frequent disease symptoms or health problems mentioned by farmers in (a) poultry farms and (b) pig farms].

Recurrent indications of disease or health complications cited by farmers in poultry and pig farms are indicated in Figure-1. In poultry farms, respiratory issues and a decline in egg production were the major issues mentioned; in pig farms, skin problems (scabies) and plagues were the fundamental health constraints.

### Knowledge about antibiotics and antibiotic resistance

In poultry, 93% of farmers testified that they were using antibiotics on their farms, while only 50% of pig farmers testified to having used antibiotics. The antibiotics commonly used on farms are listed in Table-3. Tetracyclines were most frequently used in poultry farms, followed by macrolide, polymyxin, aminoglycoside, and sulfonamide at 73%, 57%, 55%, and 50%, respectively, in the farms. In pig farms, the most frequently used antibiotic was tetracycline. About 60% of the farms that used veterinary drugs reported using it.

**Table 3:** Antibiotics used in poultry and pig farms in South Togo.

A – Antibiotics used in poultry farms (n is the total number of farms from which veterinary drugs, including antibiotics, were recorded)			

Antibiotic class	Example of antibiotic/antibiotic agent	Number of farms	Proportion (n = 113)
Tetracycline’s	Oxytetracycline; doxycycline	96	85%
Macrolide	Erythromycin/tylosin	82	73%
Polymycines	Colistin	64	57%
Aminoglycosides	Streptomycin	62	55%
Sulfonamides+inhibitor	Trimethoprim/sulfadiazine	56	50%
Furane	Furaltadone	38	34%
Quinolones	Enrofloxacin/flumequine	18	16%
Aminosides	Néomycine	16	14%
Penicillin	Penicillin G	6	5%

**B – Antibiotics used in pig farms (n is the total number of farms from which veterinary drugs, including antibiotics, were recorded)**			

**Antibiotic class**	**Example of antibiotic/antibiotic agent**	**Number of farms**	**Proportion (n = 81)**

Tetracyclines	Oxytetracycline	49	60%
Penicillin + aminoglycosides	Penicillin; streptomycine	10	12%
Quinolone	Enrofloxacin	1	1%

In general, the results demonstrated that in poultry and pig farms, 21% and 67% of breeders were unaware of antibiotics. Further, 39% and 57% did not know what antibiotic resistance was. Farmer’s knowledge assessment showed that 19% and 64% did not have adequate knowledge concerning antibiotics and antibiotic resistance in poultry and pig farms (Table-4).

**Table 4:** Knowledge of poultry and pig farmers concerning antibiotics and antibiotic resistance.

Knowledge of poultry farmers concerning antibiotics and antibiotic resistance			

	Satisfactory (n = 98/81%)	Unsatisfactory (23/19%)	p-value
Region			
Maritime	51 (72.9%)	19 (27.1%)	0.006
Plateau	47 (92.2%)	4 (7.8%)	
Gender			
Male	84 (80.0%)	21 (20.0%)	0.734
Female	14 (87.5%)	2 (12.5%)	
Education			
Non-instructed	1 (50%)	1 (50%)	0.050
Primary and secondary level	41 (73.2%)	15 (26.8%)	
University level	56 (88.9%)	7 (11.1%)	
Training			
Yes	63 (85.1%)	11 (14.9%)	0.145
No	35 (74.5%)	12 (25.5%)	
Type of farm			
Modern	73 (78.5%)	20 (21.5%)	0.202
Improved traditional farm	25 (89.3%)	3 (10.7%)	
Farm size			
Small (<1000)	69 (82.1%)	15 (17.9%)	0.764
Intermediate (1000 and 5000)	23 (76.7%)	7 (23.3%)	
Big size (>5000)	6 (85.7%)	1 (14.3%)	
Seniority of the farm			
Recent	76 (80.0%)	19 (20.0%)	0.595
Old	22 (84.6%)	4 (15.4%)	
Regularity of the production			
Permanent production	73 (83.9%)	14 (16.1%)	0.191
Temporary production	25 (73.5%)	9 (26.5%)	

**Knowledge of pig farmers concerning antibiotics and antibiotic resistance**			

	**Satisfactory (n = 34/35.1%)**	**Unsatisfactory (63/64.9%)**	**p-value**

Region			
Maritime	14 (30.4%)	32 (69.6%)	0.400
Plateau	20 (39.2%)	31 (60.8%)	
Gender			
Male	29 (43.9%)	37 (56.1%)	0.011
Female	5 (16.1%)	26 (83.9%)	
Education			
Not instructed	2 (7.4%)	25 (92.6%)	0.000
Primary and secondary level	18 (34.6%)	34 (65.4%)	
University level	14 (77.8%)	4 (22.2%)	
Training			
Yes	16 (61.5%)	10 (38.5%)	0.002
No	18 (25.4%)	53 (74.6%)	
Type of farm			
Modern	18 (66.7%)	9 (33.3%)	0.000
Improved traditional	16 (22.9%)	54 (77.1%)	
Farm size			
Small (<1000)	12 (27.9%)	31 (72.1%)	0.085
Intermediate (1000–5000)	16 (35.6%)	29 (64.4%)	
Big size (>5000)	6 (66.7%)	3 (33.3%)	
Seniority of the farm			
Recent	26 (37.7%)	43 (62.3%)	0.271
Old	8 (28.6%)	20 (71.4%)	
Regularity of the production			
Permanent production	20 (28.2%)	51 (71.8%)	0.019
Temporary production	14 (53.8%)	12 (46.2%)	

In poultry, the measure of competence was substantially linked to the region and the farmers’ education level. In pig farms, knowledge was significantly linked to gender, education level, specific training status, and regularity of production (p < 0.05). Male farmers, those with university-level education, and those who benefited from training were likelier to have sufficient knowledge regarding antibiotic and antibiotic resistance (p < 0.05).

### Farmers’ practice concerning antibiotic use

Breeders’ practices are presented in Table-2, and the relationship between farmers and farm status is presented in Table-5. Overall, the outcomes demonstrated that poultry farmers followed good practices regarding the veterinary prescription requirement before purchasing antibiotics (63%), and where antibiotics should be procured (90%). Substandard practices were linked to laboratory testing requirements (93% of farmers had never sent samples to the laboratory) and antibiotic use (82% use antibiotics for prevention). Then, all breeders who followed substandard practices were 43%. In pig farms, substandard practices were associated with nearly all the variables used to evaluate good practices – 84% of farmers used antibiotics as prophylactics, 57% of farmers procured antibiotics without veterinary prescription, 51% procured antibiotics in the local market or from other farmers, and 98% of farmers had never sent any sample to the laboratory for testing. About 61% of farmers had unacceptable practices. Substandard practices were not substantially linked to the characteristics of the breeders or farms in pig farming (p > 0.05).

**Table 5:** Practices of poultry and pig farmers concerning antibiotics and antibiotic resistance.

Practices of poultry farmers concerning antibiotics and antibiotic resistance			

	Satisfactory (n = 68/56.2%)	Unsatisfactory (n = 53/43.8%)	p-value
Region			
Maritime	42 (60%)	28 (40%)	0.357
Plateau	26 (51%)	25 (49%)	
Gender			
Male	58 (55.2%)	47 (44.8%)	0.585
Female	10 (62.5%)	6 (37.5%)	
Education			
Not instructed	2 (100%)	0 (0.0%)	0,078
Primary and secondary level	26 (46.4%)	30 (53.6%)	
University level	40 (63.5%)	23 (36.5%)	
Training			
Yes	45 (60.8%)	29 (39.2%)	0,199
No	23 (48.9%)	24 (51.1%)	
Type of farm			
Modern	54 (58.1%)	39 (41.9%)	0.517
Improved traditional	14 (50%)	14 (50%)	
Farm size			
Small (<1000)	49 (58.3%)	35 (41.7%)	0.683
Intermediate (1000–5000)	16 (53.3%)	14 (46.7%)	
Big size (SUP 5000)	3 (42.9%)	4 (57.1%)	
Seniority of the farm			
Recent	56 (58.9%)	39 (41.1%)	0.271
Old	12 (46.2%)	14 (53.8%)	
Regularity of the production			
Permanent production	45 (51.7%)	42 (48.3%)	0.154
Temporary production	23 (67.6%)	11 (32.4%)	

	**Satisfactory (n = 30/30.9%)**	**Unsatisfactory (n = 67/69.1%)**	**p-value**

**Risk practices of pig farmers concerning antibiotics and antibiotic resistance**			

Region			
Maritime	12 (26.1%)	34 (73.9%)	0.327
Plateau	18 (35.3%)	33 (64.7%)	
Gender			
Male	23 (34.8%)	43 (65.2%)	0.249
Female	7 (22.6%)	24 (77.4%)	
Education			
Not instructed	7 (25.9%)	20 (74.1%)	0.802
Primary and secondary level	17 (32.7%)	35 (67.3%)	
University level	6 (33.3%)	12 (66.7%)	
Training			
Yes	9 (34.6%)	17 (65.4%)	0.629
No	21 (29.6%)	50 (70.4%)	
Type of farm			
Modern	11 (40.7%)	16 (59.3%)	0.225
Improved traditional	19 (27.1%)	51 (72.9%)	
Farm size			
Small (<100)	10 (23.3%)	33 (76.7%)	0.333
Intermediate (entre 100 and 500)	17 (37.8%)	28 (62.2%)	
Big size (> 500)	3 (33.3%)	6 (66.7%)	
Seniority of the farm			
Recent	21 (30.4%)	48 (69.6%)	0.271
Old	9 (32.1%)	19 (67.9%)	
Regularity of the production			
Permanent production	45 (51.7%)	42 (48.3%)	0.869
Temporary Production	23 (67.6%)	11 (32.4%)	

### Typology of breeders according to their knowledge and practices

**Figure 2:**
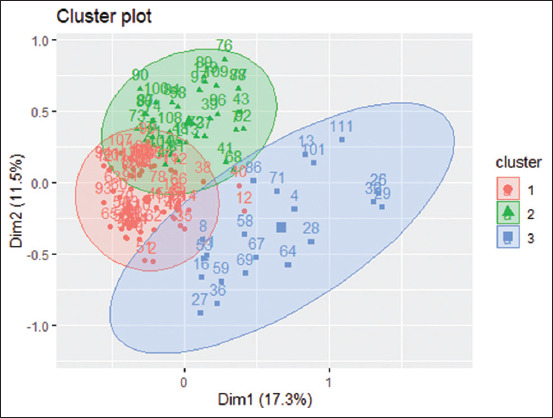
[Representation of farmers clusters on the factorial axes 1 and 2 in poultry farms; the numbers correspond to the breeders].

The MCA results for poultry farmers made it plausible to regard two axes explaining 28.8% of the total information. This permitted the projection of all breeders for classification into association groups. The factorial axis 1 was the major axis and explained 17.3% of the variability. Axis 2 explained only 11.5% of the total variability. Figure-2 demonstrates the prediction of individual farmers on the plane of the factorial axes 1 and 2, which permitted recording the affinity in three clusters or classes. Class 1, comprising 64 farmers, is characterized by good hygiene practices (100%) and good knowledge of antibiotics (100%). They were principally from the Maritime region and had contemporary farms. Class 2 accounts for 34 farmers and comprises farmers with bad hygiene practices on-farm (51%) but with competence regarding antibiotics (100%). Farmers of this class were majorly from the Plateau regions (75%) and owned modern farms (62.1%) with small sizes (86.2%). Finally, the last class involves breeders following improper hygiene at farms (53%) and insufficient knowledge about antibiotics (82.1%) and practices regarding antibiotic use (60.7%). The important modalities linked with each class can be available as supplementary material from the corresponding author.

**Figure 3:**
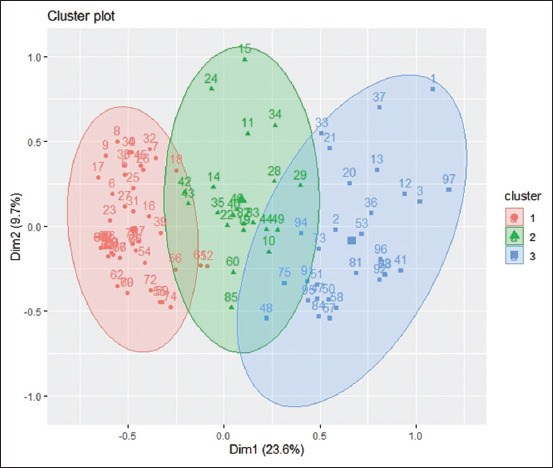
[Representation of farmers clusters on the factorial axes 1 and 2 in pig farms; the numbers correspond to the breeders].

In pig farms, 33.3% of the total variability was explained by the two dimensions considered for projecting individual farmers. The first axis explained 23.6% of the variability, while the second axis contained only 9.7% of the total information. Three clusters or classes were also defined after the projection of farmers (Figure-3). Class 1, constituting 46 pig farmers, is characterized by inadequate hygiene (100%) at the farm as well as insufficient knowledge (100%) and practices (86.05%) regarding antibiotics and antibiotic resistance. Breeders of this class were primarily women (55%) with farms of small size (60%). Unlike the precedent class, the second class comprises 21 farmers with sufficient knowledge (62.12%) and practices (54.1%) concerning antibiotic use. Farmers of this class came from the Plateau region (67.6%) and 81% were men. The third class constitutes farmers with unsatisfactory farm hygiene but a good knowledge of antibiotics and antibiotic resistance. Farmers of this class were all men (100%) and majorly from the Maritime region (88.24%) [Supplementary data can be available from the corresponding author].

## Discussion

Several factors are responsible for the increased occurrence of antibiotic resistance. Among these factors, preventive use and treatment with antibiotics are the most crucial factors. Knowledge, attitudes, and practices of livestock breeders regarding antibiotic use are also considered a substantial cause for the development of antibiotic resistance on farms [16]. This study recorded poultry and pig farmers’ competence and practices regarding antibiotics and antibiotic resistance in Togo.

Farmers were usually male (in both poultry and pig farms). Most of the poultry farmers had university-level education and the pig farmers had secondary-level education. Poultry farms were contemporary and required capital to begin, which explains the larger proportion of males compared with predominantly traditional pig farms. A similar study on pig farmers in China reported that 63% of males and 38% of females had a middle-level education. However, according to the same study, only 18% of farmers reported that they had followed the training for pig breeding. The apparent high education levels and the high fraction of trained farmers in poultry farms are due to the sensitivity required for this venture, which requires a minimum knowledge to support investments and produce profits.

The bulk of the poultry and pig farms were of small size. These features are unsurprising, given that many farms are new (<10 years old) with little investment and are run by professionals for extra income – similar to farm characteristics in Chad, studied by Bodering *et al*. [17]. In contrast to pig farms, most poultry producers used footbaths. In North Georgia, poultry growers reported a similar frequency of footbath usage (51.3%) [18]. However, in Belgium, pig farmers reported a more consistent footbath use on farms [19]. This low rate of footbath usage in pig farms found in our research could explain the diseases experienced by pig farmers and that lead to antibiotic use. Therefore, compliance with this basic biosecurity measure can prevent unnecessary antibiotic use to control infectious diseases.

In poultry farms, respiratory problems, decreased egg-laying, and diarrhea were reported as constant health issues. In addition, skin problems (scabies) and plagues (African swine fever) have been reported in pig farms. However, since laboratory testing was uncommon on most farms and breeders did not use veterinary care, these health issues were frequently treated with antibiotics as symptomatic treatment resulting in crucial misuse. These results emphasize the need to educate farmers on the relevance of veterinary care on farms. Okwelum *et al*. [20] reported similar results in Nigeria, indicating that weight loss had the greatest prevalence, followed by bloody diarrhea and respiratory issues. Concerning pig farms, mange is a health concern for pigs as reported by Laha [21]. The plague is very recent and contributes to a real health challenge to pig farming in Togo. In fact, since 2015, African swine fever has emerged in Togo and occasionally causes massive losses in pig farms, particularly in those with an unsatisfactory level of hygiene, as reported in this study.

In poultry and pig farms, tetracycline was the most commonly used antibiotic. In a survey conducted in livestock systems across five countries in Africa, authors also reported that tetracycline was the most widely used antibiotic [22]. However, research conducted in China [14] reported amoxicillin as the most widely used antibiotic there (76.5%). The accessibility of antibiotics could corroborate this apparent variation between China and Togo. Indeed, the antibiotics used by breeders in Togo are those available in the country market. Several investigations reported a similar antibiotic use pattern on poultry farms [23]. In pig farms, Lekagul *et al*. [24] found that common classes of antibiotics used differed across countries. Farmers in our study mainly use antibiotics for preventive purposes, contrary to Caudell *et al*. [22], who found that the most common reason for using antimicrobials was treatment followed by preventing sickness in groups and individual animals.

Concerning hygiene, most poultry farmers had acceptable hygiene practices, whereas only 15% of pig farmers had acceptable hygiene practices. To evaluate biosecurity objectively, it is advised to describe the practices on each farm and provide tangible management suggestions based on the assessment [25]. We attempted such a quantification method here, even if it was on a tiny scale, giving us an indication of the hygiene level on each farm. Substandard hygiene practices were observed in 75% of pig farms, suggesting a high risk of developing antibiotic resistance. However, biosecurity routines on pig farms, such as efficient cleaning and disinfection protocols, seem to impact antibiotic resistance development [26]. In contrast to Togo, an assessment carried out in developed countries revealed a high level of external biosecurity in pig farms and good internal biosecurity with a more rigorous assessment tool [27].

Regarding knowledge and practices about antibiotic resistance, 21% of poultry and 67% of pig farmers were unaware of antibiotics. A similar study in Chinese pig farms also reported that two-thirds of the participants were unaware of antibiotics [28]. In addition, antibiotic resistance was recognized by more pig farmers than by poultry farmers. Knowledge is better in poultry, where most farmers have satisfactory competence. These results should serve as caution since, on most farms, no veterinary care was used. This indicates the need for enlightenment programs for farmers. Knowledge was substantially influenced by the education levels of farmers, indicating a higher risk of antibiotic mismanagement by farmers with low or no access to formal education. In pig farms, in particular, knowledge was also linked to gender, and male farmers were likelier to have an adequate measure of knowledge. A similar study stated that male farmers had better knowledge about antibiotics in China [28].

Globally, of all poultry farmers, 43% had inadequate antibiotic usage procedures, compared with 69% of pig farmers. The fact that pig farms were, for the most part, traditionally improved farms operated by low-level educated farmers could explain the higher non-compliance with good practices.

## Conclusion

Despite the limitations of this study, which include a small sample size (the maximum number of breeders accessible and willing to participate), it provides valuable information on the level of biosecurity on farms in southern Togo, as well as breeders’ knowledge, attitudes, and practices regarding antibiotic resistance. The results demonstrated substantial non-compliance with biosecurity measures and good practices toward antibiotic use on many farms. Therefore, the probability that antibiotic resistance could develop in the food-producing animals is alarmingly high, and action is needed, especially regarding farmers’ responsibility in animal production. These findings can be used to facilitate training and sensitize farmers. However, as production conditions vary greatly due to socioeconomic, political, and environmental factors, regulations applied in other parts of the world, especially in livestock exporting countries, should be adapted for the developing countries, and methods should be found to educate and equip farmers [29]. This could be a way to enhance the knowledge and practices of breeders for enhanced and responsible animal production.

## Data availability

Supplementary data can be available from the corresponding author on a reasonable request.

## Authors’ Contributions

APB and RBA: Conceptualized and directed the work. APB and MD: Collected data from the field. ET, MS, MOS, and KFD. Analyzed the data. APB: Drafted the manuscript. All authors have revised the manuscript. All authors have read and approved the final submission.
